# Nonalcoholic Fatty Liver Disease and Coronary Artery Calcification in a Northern Chinese Population: a Cross Sectional Study

**DOI:** 10.1038/s41598-017-09851-5

**Published:** 2017-08-30

**Authors:** Rina Wu, Feng Hou, Xiaomin Wang, Yong Zhou, Kai Sun, Youxin Wang, Henghui Liu, Jing Wu, Ruiping Zhao, Jiang Hu

**Affiliations:** 1 Department of Cardiology, Central Hospital of Baotou, Baotou, 014040 China; 2 Graduate school, Inner Mongolia Medical University, Hohhot, 010000 China; 3 Department of Cardiology, Beijing An Zhen Hospital, Capital Medical University, Beijing Institute of Heart, Lung and Blood Vascular Diseases, Beijing, 100050 China; 4 Department of Radiology, Central Hospital of Baotou, Baotou, 014040 China; 5 Beijing Key Laboratory of Clinical Epidemiology, School of Public Health, Capital Medical University, Beijing, 100069 China; 6 Beijing Recdata Technology Co., Ltd, Beijing, 100050 China; 7 Department of Surgery, Central Hospital of Baotou, Baotou, 014040 China

## Abstract

Nonalcoholic fatty liver disease (NAFLD) has become an emerging health issue with a high prevalence in general population. The cross-sectional study was performed to investigate the association between NAFLD and coronary artery calcification (CAC) in individuals from northern city of China. A total of 2345 participants aged ≥40 (1035 men and 1310 women) were selected from the Jidong community of Tangshan city. Liver ultrasonography was used to the diagnosis of NAFLD. A 64-slice CT scanner was used to determine coronary artery calcification score (CACS), with CACS > 0 defined to be the presence of CAC. The risk level of coronary heart disease (CHD) was graded by CACS according to the 4 commonly used thresholds in clinical practice (0, 10, 100, and 400 Agatston units). NAFLD was significantly associated with CAC (crude OR: 1.631, 95% CI: 1.295–2.053, adjusted OR: 1.348, 95% CI: 1.030–1.765). The association between NAFLD and increased risk level of CHD (Crude OR: 1.639 95% CI: 1.303–2.063; adjusted OR: 1.359 95% CI: 1.043–1.770) was observed. The associations between NAFLD and CAC or increased risk level of CHD were significant in female but not in male. Our finding further confirmed the association between NAFLD and CAC, especially in Asian population.

## Introduction

According to the report of the World Health Organization (WHO) in 2012, as a leading cause of morbidity and mortality worldwide which is a main result of coronary atherosclerosis, the CHD has been the most serious global health issue^1^. In order to prevent or delay the progression of CHD, early detection of CAC status for asymptomatic individuals is required. The coronary artery calcification score (CACS) detected by multi-slice computed tomography (MSCT), as a reliable marker of subclinical atherosclerosis has been widely used to predict the cardiovascular diseases^2, 3^. Polonsky *et al*.^4^ suggested that addition of CACS to the predicting model based on traditional risk factors significantly improved the classification of risk. In addition, CACS was showed to be associated with the development of coronary artery disease and the risk of future cardiovascular events in large prospective studies^5, 6^.

Recently, nonalcoholic fatty liver disease (NAFLD) has become an emerging health problem with a prevalence of up to 30% in general population^7^. Some studies suggested that NAFLD was a manifestation of the metabolic syndrome (MS) in the liver, and its pathological feature was abnormal deposition of lipid in liver cells^8^. According to the result of a longitudinal cohort study, patients with NAFLD were found to have a higher mortality due to CHD than the liver cirrhosis^9^. Moreover, a series of previous studies suggested that NAFLD is associated with CHD, independent of traditional risk factors and other metabolic factors^10–13^. Therefore it can be hypothesized that NAFLD may associate with subclinical atherosclerosis determined by CAC.

We aimed to explore the association between NAFLD and CAC in a cross-sectional study conducted in a northern Chinese cohort consisting of 2,345 adults who have no history and clinical symptoms of cardiovascular disease.

## Result

**Figure 1:**
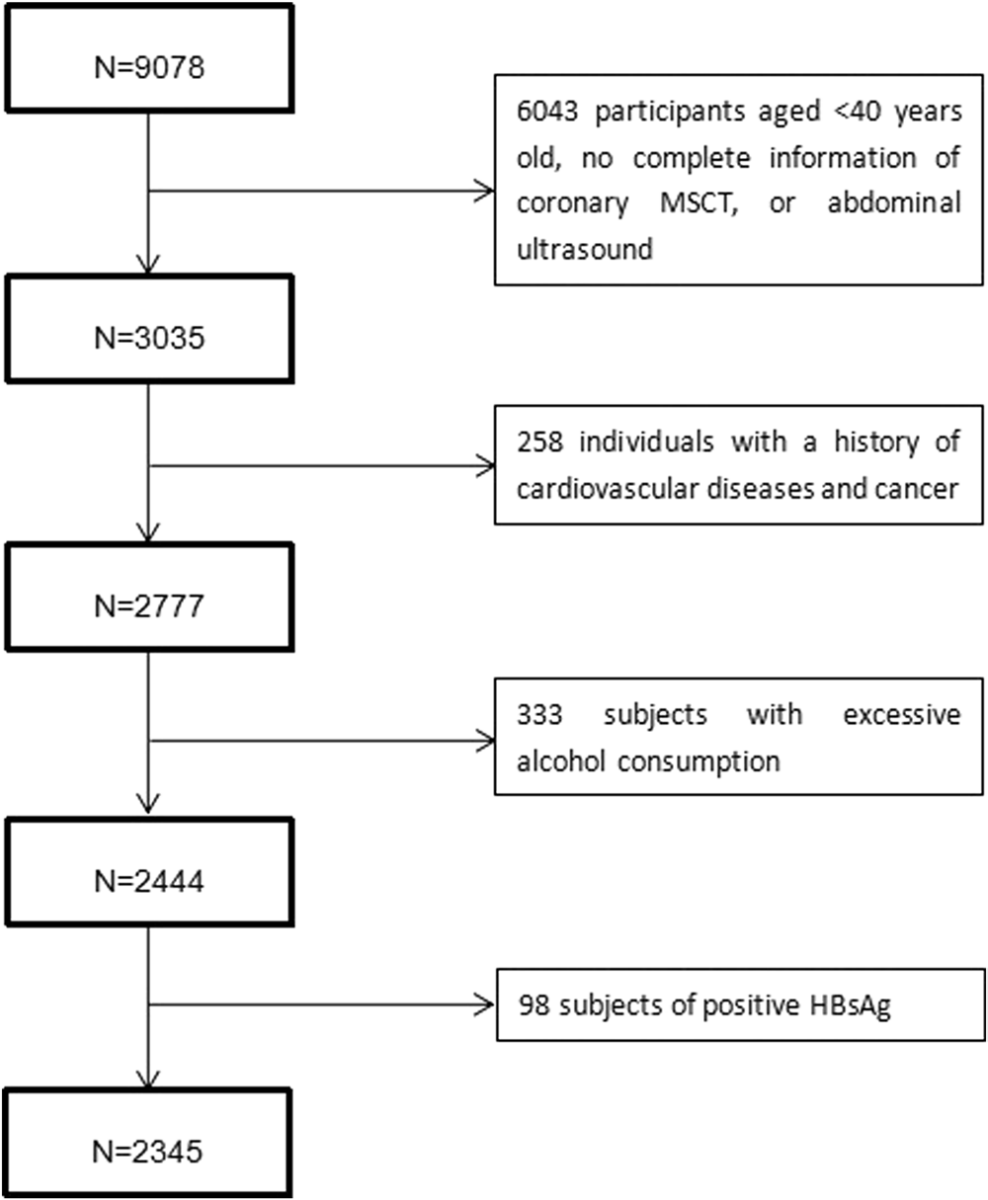
Flow Chart of the Enrolled Participants Meet the Requirements.

Figure 1 showed the flow chart for the subject selection under different exclusion criteria. In total 9078 subjects were enrolled and finally 2345 were satisfied the conditions of the present study.

The characteristics of study participants were shown in Table 1. The mean age was 55.65 ± 7.79 and 44.14% (n = 1035) were man. About 15.74% (n = 369) of participants had CAC, with 59.07% (n = 218) in male and 40.92% (n = 151) in female. 21.11% (n = 495) were current smokers. Participants with CAC were of low or moderate level of education, and lower average income. Participants in the CACS > 0 group were older, had higher BMI, and tended to have a higher percentage of NAFLD, diabetes mellitus, hypertension, hyperlipidemia. In addition, parameters including blood pressure (both SBP and DBP), FBG, TG, TC, LDL, and Cr, were higher in participants with presence of CAC, while HDL level was lower in participants with presence of CAC, compared these with absence of CAC (CACS = 0). There was no significant difference between the group with CAC and without CAC in the marital status and level of AST or ALT.

**Table 1:** Characteristics of Participants Stratified by the Status of CAC

Characters	Total (n = 2345)	CACS		*P* value
		CACS = 0 (n = 1976)	CACS > 0 (n = 369)	
**Gender n(%)**				<0.001
Men	1035(44.14)	817(78.94)	218(21.06)	
Women	1310(55.86)	1159(88.47)	151(11.53)	
Age, years (x ± s)	55.66 ± 7.79	54.65 ± 7.55	61.05 ± 6.77	<0.001
**Education level n(%)**				<0.001
Illiterate or primary	213(9.08)	158(74.18)	55(25.82)	
Middle/high school	1467(62.56)	1217(82.96)	250(17.04)	
College or above	665(28.36)	601(90.38)	64(9.62)	
Income, ¥/month n(%)*				0.001
<3000	1306(55.70)	1069(81.85)	237(18.15)	
3001~5000	901(38.42)	782(86.79)	119(13.21)	
≧5001	109(4.65)	100(91.74)	9 (8.26)	
**Smoking n(%)**				<0.001
Never	1743(74.33)	1510(86.63)	233(13.37)	
Current smoker	495(21.11)	393(79.39)	102(20.61)	
Former smoker	107(4.56)	73(68.22)	34(31.78)	
**Physical activity n(%)**				0.021
Inactive	707(30.15)	601(85.01)	106(14.99)	
Moderately active	161(6.87)	147(91.30)	14(8.70)	
Very active	1477(62.98)	1228(83.14)	249(16.86)	
BMI (kg/m^2^)	25.09 ± 3.43	24.95 ± 3.32	25.83 ± 3.92	<0.001
AC (cm)	86.95 ± 9.91	86.24 ± 9.81	90.76 ± 9.63	<0.001
HC (cm)	98.61 ± 7.30	98.46 ± 7.27	99.43 ± 7.37	0.019
SBP (mmHg)	133.17 ± 20.26	131.90 ± 19.72	140.20 ± 21.64	<0.001
DBP (mmHg)	83.76 ± 13.08	83.46 ± 13.13	85.36 ± 12.72	0.011
FBG(mg/dL)	5.57 ± 1.41	5.51 ± 1.32	5.93 ± 1.75	<0.001
TG (mmol/L)	1.74 ± 1.40	1.71 ± 1.40	1.88 ± 1.41	0.045
TC (mmol/L)	4.93 ± 0.94	4.90 ± 0.90	5.10 ± 1.14	0.001
HDL (mmol/L)	1.22 ± 0.28	1.23 ± 0.28	1.18 ± 0.27	0.001
LDL (mmol/L)	2.70 ± 0.61	2.67 ± 0.61	2.83 ± 0.64	<0.001
ALT (U/L)	22.80 ± 17.71	22.87 ± 17.53	22.40 ± 18.64	0.637
AST (U/L)	24.24 ± 11.26	24.34 ± 11.47	23.72 ± 10.05	0.369
Cr (umol/L)	76.96 ± 19.39	76.51 ± 19.77	79.38 ± 17.02	0.004
NAFLD n(%)	1272(54.24)	1035(81.37)	237(18.63)	<0.001
Diabetes n(%)	290(12.37)	214(73.79)	76(26.21)	<0.001
Hypertension n(%)	1096(46.74)	862(78.65)	234(21.35)	<0.001
Hyperlipidemia n(%)	1218(51.94)	988 (81.12)	230(18.88)	<0.001

Values are expressed as mean ± standard deviation, median (interquartile range) or percentage.

CACS, coronary artery calcification score; BMI, body mass index; AC, Abdomen circumference; HC, Hip circumference; DBP, diastolic blood pressure; SBP, systolic blood pressure; FBG, Fasting blood glucose; TG, Triglycerides; TC, Total cholesterol; AST, aspartate aminotransferase; ALT, alanine aminotransferase; HDL, high-density lipoprotein; Cr, serum Creatinine; NAFLD, nonalcoholic fatty liver disease.

*29 people didn’t registered the income levels information.

**Figure 2:**
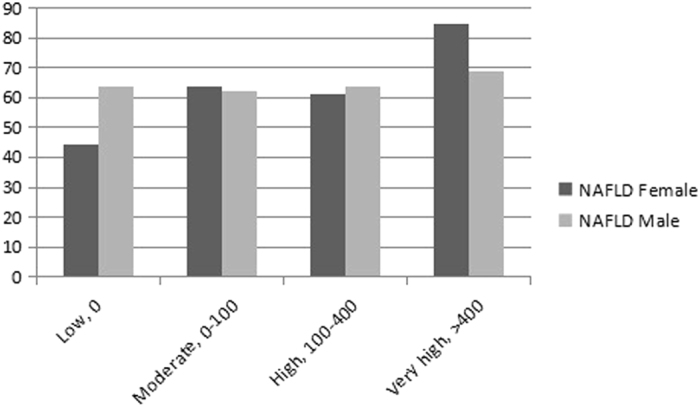
The prevalence of NAFLD in Jidong Community by sex and Risk Level of CHD.

Figure 2 showed the prevalence of NAFLD in different CHD risk levels, stratified by gender. The prevalence of NAFLD in males were 63.89%, 62.39%, 63.77%, 68.75% in four categories of risk for CHD, respectively, while in female are 44.26%, 63.92%, 60.98%, 84.62% in four categories of risk for CHD, respectively. The proportions seem to have an increasing trend with the increased risk level of CHD, no matter for male or female.

Table 2 showed the Odds Ratio (95% CI) of presence of NAFLD and CAC (CACS > 0), among all the subjects, together with these stratified by sex. In order to investigate whether NAFLD is independently associated with CAC, we adjusted age and gender in model 2, age, gender, smoking status, hypertension and diabetes in model 3, and additionally adjusted HC, LDL, physical activity, level of education and income on the basis of model 3 in model 4. For all participants, statistical significances were observed in crude model and other 3 models adjusted confounding factors including sociological features and traditional risk factors (Crude model: OR: 1.632, 95% CI: 1.296–2.055, p < 0.001; Model 2: OR: 1.490, 95% CI: 1.165–1.905, p = 0.002; Model 3: OR: 1.332, 95% CI: 1.034–1.715, p = 0.027; Model 4: OR: 1.348, 95% CI: 1.030–1.765, p = 0.030). Furthermore, we observed that this association was more significant in female than these in male (OR: 1.568 95% CI: 1.072–2.293, p = 0.020 vs. OR: 1.143 95% CI: 0.812–1.609, p = 0.443).

**Table 2:** Multivariable Analyses of Relationship between NAFLD and CAC Stratified by Sex.

	Presence of CAC		*P* value	Increased risk of CHD		*P* value
	No NAFLD	NAFLD		No NAFLD	NAFLD	
Total						
Model 1	1	1.632 (1.296, 2.055)	<0.001	1	1.639 (1.303, 2.063)	<0.001
Model 2	1	1.490 (1.165, 1.905)	0.002	1	1.479 (1.162, 1.884)	0.002
Model 3	1	1.332 (1.034, 1.715)	0.027	1	1.326 (1.034, 1.700)	0.026
Model 4	1	1.348 (1.030,1.765)	0.030	1	1.359 (1.043, 1.770)	0.023
Male*						
Model 1	1	0.994 (0.729,1.357)	0.972	1	1.005 (0.738, 1.369)	0.973
Model 2	1	1.228 (0.880, 1.713)	0.227	1	1.217 (0.879, 1.684)	0.236
Model 3	1	1.143 (0.812, 1.609)	0.443	1	1.130 (0.810, 1.576)	0.471
Model 4	1	1.209 (0.839, 1.742)	0.308	1	1.222 (0.856, 1.745)	0.270
Female*						
Model 1	1	2.328 (1.635, 3.317)	<0.001	1	2.337 (1.641, 3.326)	<0.001
Model 2	1	1.837 (1.271, 2.655)	0.001	1	1.856 (1.288, 2.675)	0.001
Model 3	1	1.568 (1.072, 2.293)	0.020	1	1.597 (1.096, 2.329)	0.015
Model 4	1	1.490 (0.993, 2.237)	0.054	1	1.526 (1.020, 2.281)	0.040

OR odds ratio, CI confidence interval, DM diabetes mellitus.

Model 1: without any adjusted

Model 2: adjusted for age and gender

Model 3: model 2 with additional adjustment for smoking status, Hypertension, and Diabetes

Model 4: model 3 with additional adjustment for HC, LDL, physical activity, level of education and income.

*Gender was excluded from potential adjustment factors.

Meanwhile, the statistically significant result shows that the presence of NAFLD was significantly associated with the increased risk level of CHD (Crude OR: 1.639, 95% CI: 1.303–2.063, p < 0.001). After adjustment for potential confounders in study, similar results were observed (Model 2 adjusted age and gender: OR: 1.479, 95% CI: 1.162–1.884, p = 0.001; Model 3: adjusted smoking status, hypertension and diabetes on the basis of model 2, OR: 1.326, 95% CI: 1.034–1.700, p = 0.026; Model 4: OR: 1.359, 95% CI: 1.043–1.770, p = 0.023). Similarly, this association was also significant in women than in men (OR: 1.597 95% CI: 1.096–2.329, p = 0.015 vs. OR: 1.130, 95% CI: 0.810–1.576, p = 0.471).

The characters of female participants about two groups, without menopause and with menopause, were showed in Table 3. Compared with the premenopausal group (n = 508), the women in the postmenopausal group (n = 802) had higher BMI, HC, TG, TC, and LDL. Moreover, the percentage of hypertension, diabetes, hyperlipidemia, NAFLD, and CAC were higher in the postmenopausal group.

**Table 3:** Characteristics of Female Participants Stratified by Menopause Status

Characters	Total (n = 1310)	Menopause Status		*P* value
		Premenopausal (n = 508)	Postmenopausal (n = 802)	
BMI (kg/m^2^)	24.69 ± 3.52	23.92 ± 3.48	25.17 ± 3.46	<0.001
AC (cm)	83.72 ± 9.69	80.34 ± 9.27	85.85 ± 9.34	<0.001
HC (cm)	97.49 ± 7.53	96.49 ± 6.75	98.13 ± 7.92	<0.001
TG (mmol/L)	1.58 ± 1.14	1.41 ± 1.02	1.69 ± 1.19	<0.001
TC (mmol/L)	5.02 ± 0.97	4.74 ± 0.86	5.20 ± 0.99	<0.001
LDL(mmol/L)	2.73 ± 0.64	2.53 ± 0.60	2.85 ± 0.63	<0.001
Diabetes n(%)	140(10.69)	23(16.43)	117(83.57)	<0.001
Hypertension n(%)	544(41.53)	148(27.21)	396(72.79)	<0.001
Hyperlipidemia n(%)	618(47.18)	172(27.83)	446(72.17)	<0.001
NAFLD n(%)	611(46.64)	177(28.97)	434(71.03)	<0.001
CAC n(%)				<0.001
CACS = 0	1159(88.47)	490(42.28)	669(57.72)	
CACS > 0	151(11.53)	18(11.92)	133(88.08)	

Values are expressed as mean ± standard deviation, median (interquartile range) or percentage.

CAC, coronary artery calcification; CACS, coronary artery calcification score; NAFLD, nonalcoholic fatty liver disease; BMI, body mass index; AC, Abdomen circumference; HC, Hip circumference; TG, Triglycerides; TC, Total cholesterol; LDL, Low-density lipoprotein.

## Discussion

In this study, we focused on the association between NAFLD and CAC, and our main findings are as follows: 1) NAFLD was associated with CAC or increased risk level of CHD independent of the traditional risk factors for CHD in Chinese adults. 2) The association between NAFLD and CAC or increased risk level of CHD was significant in female but not in male.

According to previous studies, MS has been demonstrated to contribute to the presence and progression of CAC^14^. As a manifestation of the MS in the liver, NAFLD was associated with increased prevalence of MS^15^. Therefore some studies on the association between NAFLD and CAC were reported, but the results were not consistent. Chen *et al*. (2010) and Chhabra *et al*. reported that NAFLD was associated with moderate to high risk of CHD (CACS > 100) in asymptomatic subjects from Taiwan and Missouri^12, 16^. Kim *et al*. (2012) reported that patients with NAFLD or abdominal obesity were at increased risk for coronary atherosclerosis after adjustment for classical coronary risk factors (OR: 1.34, 95% CI: 1.14–1.58)^17^. Other studies didn’t find the promoting effect of NAFLD on CAC and proposed that obesity attenuates the relationship between NAFLD and subclinical atherosclerosis^18, 19^, however, the sample was relative small (n = 219) or the subjects only included the White and the Black. Targher G *et al*. (2007) and Kwak MS *et al*. (2015) suggested that presence of NAFLD is associated with CAC in subjects with DM^11, 20^, whereas McKimmie *et al*. did not found the relationship between fatty liver disease and CVD in diabetic patients^21^. Moreover, a series of recent researches hold the same view that presence of NAFLD is associated with CAC independent of the traditional risk factors, metabolic syndrome and insulin resistance (IR)^22–24^.

Similarly, same to CACS, the carotid intima-media thickness (CIMT) is also used to represent the atherosclerosis status^25^. According to a meta-analysis of 37,197 asymptomatic subjects, CIMT each increase in 0.1mm, the risk of myocardial infarction increased by 10% to 15%, and the risk of stroke increased by 13% to 18%^26^. Presence of NAFLD has been shown to be significantly associated with increased CIMT (OR, 1.91; 95% CI, 1.17–3.01; p = 0.009)^27^. Park HE *et al*. investigated the relationship between NAFLD and CAC development in a longitudinal study, and suggested that NAFLD plays an important role in the occurrence of CAC, but not in the progression of CAC^28^. Sung KC *et al*. (2016) recently reported that the combination of fatty liver, IR, and obesity is associated with progression of atherosclerosis independently of DM or other cardiovascular risk factors^29^. At present, there is no consistent conclusion about the correlation between NAFLD and CAC, and further researches evaluating the role of NAFLD on cardiovascular diseases are needed in different races.

Based on a recent epidemiological report, the prevalence of NAFLD in China is lower than the estimates in developed countries, but it still reaches the epidemic proportions, and its prevalence is increasing^30^. Therefore, we should pay enough attention to the NAFLD patients who have no symptoms of CHD. Similar result with previous studies was found that it does exist a strong association of NAFLD increased the prevalence of CAC^12, 16, 17^. Besides, after adjusting for potential confounds (sex, age, hypertension, DM, smoking status, hip circumference, LDL, income and education level), we found that patients with NAFLD had higher risk of the increased risk level of CHD, compared with those who have no NAFLD, which was supported by Kim D *et al*. (2012)^17^. Our finding that NAFLD associated with CAC in Chinese population might further confirm the association between NAFLD and CAC, especially in Asian populations.

The association between NAFLD and CAC or increased risk level of CHD were significant in female but not in male might be due to the effect of menopause on increased of visceral fat and inflammation which can increase the prevalence of NAFLD^31, 32^. Consistent with previous reports, which estrogen deficiency affects body fat distribution, which increases accumulation of gluteofemoral fat and central fat, we find that estrogen may have a protective effect on NAFLD, and menopause may be the reason for increasing prevalence of NAFLD^33, 34^. In male participants, we did not get a statistically significant result of two above association, which might be explained by a presumption that the negative effect of NAFLD such as MS, IR, dyslipidemia and so on is more significant in women than in man. This result was confirmed by a recent study that suggested that fatty liver disease was a useful predictor of atherosclerosis especially for female^35^. The gender differences in association between NAFLD and CAC or increased risk level of CHD need be further investigated.

At present, the pathogeneses that connect NAFLD and CAC have not been thoroughly investigated. The previous studies suggested that NAFLD may affect the CAC in pathways including IR, oxidative stress and systemic inflammation, visceral fat, dyslipidemia and ectopic adipose tissue distribution, reduction of adiponectin, and endothelial dysfunction^36^. IR often associates with NAFLD, obesity, and DM at the same time, and it can speculate that IR plays an important role in pathogenesis of NAFLD, MS, and atherosclerosis^37–39^. An increase in lipolysis in peripheral fat tissue and subsequent elevated serum concentration of free fatty acids (FFAs) were induced by IR, resulting in the triglyceride accumulation in the liver^40, 41^. A Multi-Ethnic Study of Atherosclerosis community population showed that IR was associated with CAC incidence and progression^42^. The combination of IR and systemic inflammation may aggravate the abnormal lipid metabolism and affect the atherogenic lipid profile^28^. Additionally, oxidative stress, a critical factor in NAFLD pathogenesis which was considered to associate with severity of NAFLD, is another potential pathway which contributed to CAC^36, 43, 44^. Visceral adipose tissue (VAT), associated with visceral adiposity, can secrete pro-inflammatory cytokines, adipokines and hormones, and prove to be an effect on atherosclerosis^17, 36, 45^. Adiponectin has been demonstrated to have a role in inhibiting the secretion of cytokines from adipocytes. Reduction of plasma adiponectin levels may be associated with IR, T2DM, and obesity, and contribute to the development of preclinical atherosclerosis^46, 47^. In our participants, the proportion of patients with diabetes and dyslipidemia is relatively high (about 12.35% and 51.87%, respectively), and the BMI of the whole population is in the critical value of the obesity standard (25.09 ± 3.43). These results may suggest that NAFLD contribute to the risk of CHD by obesity, ectopic fat distribution and other diseases caused by metabolic disorders.

There are several limitations in our study. First, our study is across-sectional study so that we were not able to evaluate the causality between NAFLD and CAC. Secondly, ultrasonography was used in the diagnosis of NAFLD in our study, which has limited sensitivity^48^. As a result, our results may not be generalized to the fatty liver that cannot be recognized by ultrasonography, and we cannot rule out the influence of the experience of the physician who made the diagnosis. However, compared with other methods, ultrasonography is widely used in most studies because of its low cost, easy to implement, non-invasive, and relative accuracy. In addition, due to the most accurate classification of the severity of NAFLD is based on histological assessment that was not preformed, we do not analyze the relationship between CAC and different severity of NAFLD^49^. Thirdly, we focused on participants aged ≥40 years in this study and our subjects were at a relative high level of income status and education level compared to the general Chinese population. Therefore, our findings may need further validation in other population.

In conclusion, we observed that NAFLD was associated with CAC in northern Chinese adults, independent of traditional risk factors for CHD including gender, age, smoking, hypertension, and DM. The association between NAFLD and CAC or increased risk level of CHD were significant in female but not in male. Our finding that NAFLD associated with CAC in Chinese population further confirmed the association between NAFLD and CAC, especially in Asian populations, and suggested that NAFLD contribute to the risk of CHD by obesity, ectopic fat distribution and other diseases caused by metabolic disorders.

## Methods

### Ethics Statement

In accordance with the ethical guidelines of the declaration of Helsinki and China’s regulations and guidelines on good clinical practice, the study was approved by the Ethics Committee of the Jidong Oilfield Hospital. Before the start of the study, we got the written informed consents of all participants.

### Study design and participants

Individuals were recruited from the Jidong Community (Tangshan City, Northern China) which mainly comprised employees of the Jidong Co. Ltd. and their family members. From July 2013 to August 2014, a total of 9078 residents aged 20 years and older were invited to participate in the study at the time of their regular annual physical examination performed at the Jidong Oilfield Hospital. Residents who are willing to provide informed consents were included in the study. Among 9,078 participants, 3,035 subjects aged 40 years and above, who had complete information of coronary MSCT, abdominal ultrasound and medical history, were selected and 258 individuals who had a history of cardiovascular disease (including myocardial infarction, heart failure, atrial fibrillation or any other heart disease), stroke, and cancer, 333 subjects with excessive alcohol consumption (≥20 g/day for men and 10 g/day for women for more than a year) and 99 with positive HbsAg were excluded. Finally 2,345 participants were included in the analysis (Fig. 1).

### Assessment of demographic variables and laboratory parameters

A validated questionnaire specifically designed for this study was used to collect clinical data from all participants by trained doctors^50^. Demographic information (e.g.: age, gender, household income, level of education, marital status, smoking status, physical activity, and history of diseases) of all participants was collected. The average monthly income was reported as: ≤ ¥ 3,000; ¥ 3,000–5,000; and ≥ ¥ 5,001. The level of education was classified into: illiterate or primary, middle/high school, or college or above. Smoking status was categorized as: never, former, or current. The classification of physical activity was according to the following three kinds of circumstances: 1) inactive, almost none; 2) moderately active, 1–149 min/ week of moderate intensity or 1–74 min/week of vigorous intensity; or 3) very active, ≥150 min/week of moderate intensity or ≥75 min/week of vigorous intensity. The questionnaire also contains the history of disease, including hypertension, diabetes, hyperlipidemia, atrial fibrillation/flutter, heart failure, myocardial infarction, stroke, cancer, gout, fracture and the like. Hyperlipidemia was defined as a presence of history, using of cholesterol lowering medicine, a total cholesterol level >220 mg/dL, triglycerides >150 mg/dL, or low density lipoprotein (LDL) >160 mg/dL. People who have a self-reported history, current treatment with insulin, oral hypoglycemic agents or fasting blood glucose level >126 mg/dl was defined as diabetes mellitus (DM).

Height, weight, abdomen circumference (AC), hip circumference (HC) and blood pressure of each participant was respectively measured by trained nurses with a uniform approach and standard. Blood pressure, contain systolic blood pressure (SBP) and diastolic blood pressure (DBP), was measured using a mercury sphygmomanometer following 5 minutes of rest in a seated position. The average value of the two measurements was used to analyze. If the two measurements differed by more than 5 mmHg, an additional reading was taken, and the average of the three readings was used. Hypertension was defined as presence of at least one of the following status: 1) history of arterial hypertension; 2) using antihypertensive medication; or 3) a SBP >140 mmHg or a DBP >90 mmHg. The body mass index (BMI) was calculated as body weight (kg) divided by the square of the height (m^2^), after measuring body weight (kg) and height (cm) on the day of tests^50^.

Venous blood samples were collected in the morning by trained professional doctors from all participants after fasting for at least 12 hours. Vacuum tubes containing EDTA (Ethylene Diamine Tetra Acetic Acid) were used to store blood sample. After that, automated analyzers (Olympus, AU400, Japan) were used to analyze samples in the central laboratory in Jidong Oilfield Hospital^50^. In the study, many biological indicators were included such as fasting blood glucose (FBG), total cholesterol (TC), serum triglycerides (TG), serum high-density lipoprotein (HDL) cholesterol, Low-density lipoprotein (LDL) cholesterol, aminotransferase (AST), alanine aminotransferase (ALT), and serum Creatinine (Cr).

### Ultrasonographic Examinations

According to a recent meta-analysis of 49 studies with ultrasound and liver histology, the detection of fatty liver by ultrasound is considered as an accurate, reliable imaging technique compared with histology, with a pooled sensitivity of 84.8% and a pooled specificity of 93.6% for detecting ≥20% to 30% steatosis^29, 48^. Abdominal ultrasonography was performed by two experienced radiologists, who were blinded to clinical presentation and laboratory findings of participants. Liver ultrasonography, examined by a high-resolution B-mode topographic ultrasound system with a 3.5 MHz probe (ACUSON ×300, Siemens, Germany), was used to diagnose fatty liver. According to the Asia-Pacific Working Party on NAFLD and Chinese Association for the Study of Liver Disease (CASLD), diagnosis of fatty liver disease was based on the presence of at least two of the following three abnormal findings: (1) diffusely increased liver near field ultrasound echo (‘bright liver’); (2) liver echo greater than kidney; (3) vascular blurring and the gradual attenuation of far field ultrasound echo^51, 52^.

### Measurement of CAC by Multi-Slice CT

The measurement of CAC was performed by a 64-slice CT scanner (Somatom Sensation 64; Siemens Medical Solutions, Germany) with a uniform standard process. Two experienced and trained CT analysts completed image reconstruction and analysis using semiautomatic software (syno Multi-Modality Workplace, syngo MMWP VW 40 A, Siemens, Germany). Then, CACS was the sum of calcified plaque scores in all coronary arteries which was separately calculated quantitatively according to the Agatston method^53^. The presence of CAC was defined as CACS > 0, and the risk level of CHD was defined by CACS according to the 4 commonly used thresholds in clinical practice (0, 10, 100, and 400 Agatston units)^4, 54^. Due to the relative small number of subjects in the group of the 0–10, we merged it with the 10–100, and finally categorized into 4 categories: low (CACS = 0), moderate (0 < CACS ≤ 100), high (100 < CACS ≤ 400), and very high (CACS > 400)^4, 55^.

### Statistical Analyses

All data were handled and managed by using the Ruichi Precision Medical Record System (RPMRS), which was developed to standardize, integrate, manage, and analyze precision medical data. The statistical analyses were performed using SAS software (version 9.4; SAS Institute, Cary, North Carolina, USA). Continuous variables were shown as mean (standard deviation, SD) and compared using analysis of variance (ANOVA) or T-test. Categorical variables were described with percentages and compared using Chi-square test and ordinal variables were non-parameter test. Multinomial logistic regression was used to explore the association between NAFLD and presence of CAC by calculating the odds ratios (ORs) and 95% confidence interval (CI) after controlling potential confounders. We also investigated the association between NAFLD with the different level of increased CACS. Covariates with clinical importance were included in the multivariable model, such as age, gender, education level, income level, smoking status, physical activity, LDL cholesterol, HC, and diseases including hypertension and diabetes all of which were considered as risk of CHD. These associations were also investigated in stratification of gender. The statistical significance were set at α = 0.05 (two-tailed).

## References

[1] Mendis, S. *et al*. Global atlas on cardiovascular disease prevention and control. *Geneva World Health Organization* (2012).

[2] Greenland P (2010). ACCF/AHA guideline for assessment of cardiovascular risk in asymptomatic adults: a report of the American College of Cardiology Foundation/American Heart Association Task Force on Practice Guidelines. J Am Coll Cardiol.

[3] Raggi P (2008). Coronary artery calcium to predict all-cause mortality in elderly men and women. J Am Coll Cardiol.

[4] Polonsky TS (2010). Coronary artery calcium score and risk classification for coronary heart disease prediction. JAMA.

[5] Budoff MJ (2007). Long-term prognosis associated with coronary calcification: observations from a registry of 25,253 patients. J Am Coll Cardiol.

[6] Budoff MJ (2010). Progression of coronary artery calcium predicts all-cause mortality. JACC Cardiovasc Imaging.

[7] Chalasani N (2012). The diagnosis and management of non-alcoholic fatty liver disease: practice Guideline by the American Association for the Study of Liver Diseases, American College of Gastroenterology, and the American Gastroenterological Association. Hepatology.

[8] Targher G, Marra F, Marchesini G (2008). Increased risk of cardiovascular disease in non-alcoholic fatty liver disease: causal effect or epiphenomenon?. Diabetologia.

[9] Ekstedt M (2006). Long-term follow-up of patients with NAFLD and elevated liver enzymes. Hepatology.

[10] Lee YH (2011). The severity of Fatty liver disease relating to metabolic abnormalities independently predicts coronary calcification. Radiol Res Pract.

[11] Targher G (2007). Nonalcoholic fatty liver disease is independently associated with an increased incidence of cardiovascular events in type 2 diabetic patients. Diabetes Care.

[12] Chhabra R (2013). Association of coronary artery calcification with hepatic steatosis in asymptomatic individuals. Mayo Clin Proc.

[13] Lee MK (2015). Higher association of coronary artery calcification with non-alcoholic fatty liver disease than with abdominal obesity in middle-aged Korean men: the Kangbuk Samsung Health Study. Cardiovasc Diabetol.

[14] Kim LK (2016). Impact of metabolic syndrome on the progression of coronary calcium and of coronary artery disease assessed by repeated cardiac computed tomography scans. Cardiovasc Diabetol.

[15] Kim JY (2016). Relationship between non-alcoholic fatty liver disease, metabolic syndrome and insulin resistance in Korean adults: A cross-sectional study. Clin Chim Acta.

[16] Chen CH, Nien CK, Yang CC, Yeh YH (2010). Association between nonalcoholic fatty liver disease and coronary artery calcification. Dig Dis Sci.

[17] Kim D (2012). Nonalcoholic fatty liver disease is associated with coronary artery calcification. Hepatology.

[18] Ding J (2008). Association between non-subcutaneous adiposity and calcified coronary plaque: a substudy of the Multi-Ethnic Study of Atherosclerosis. Am J Clin Nutr.

[19] VanWagner LB (2014). Associations between nonalcoholic fatty liver disease and subclinical atherosclerosis in middle-aged adults: the Coronary Artery Risk Development in Young Adults Study. Atherosclerosis.

[20] Kwak MS (2015). Nonalcoholic fatty liver disease is associated with coronary artery calcium score in diabetes patients with higher HbA1c. Diabetol Metab Syndr.

[21] McKimmie RL (2008). Hepatic steatosis and subclinical cardiovascular disease in a cohort enriched for type 2 diabetes: the Diabetes Heart Study. Am J Gastroenterol.

[22] Al Rifai M (2015). The association of nonalcoholic fatty liver disease, obesity, and metabolic syndrome, with systemic inflammation and subclinical atherosclerosis: the Multi-Ethnic Study of Atherosclerosis (MESA). Atherosclerosis.

[23] Mellinger JL (2015). Hepatic steatosis and cardiovascular disease outcomes: An analysis of the Framingham Heart Study. J Hepatol.

[24] Sung KC (2013). Arterial stiffness, fatty liver and the presence of coronary artery calcium in a large population cohort. Cardiovasc Diabetol.

[25] Nambi V (2012). Common carotid artery intima-media thickness is as good as carotid intima-media thickness of all carotid artery segments in improving prediction of coronary heart disease risk in the Atherosclerosis Risk in Communities (ARIC) study. Eur Heart J.

[26] Lorenz MW (2007). Prediction of clinical cardiovascular events with carotid intima-media thickness: a systematic review and meta-analysis. Circulation.

[27] Lankarani KB (2013). Common carotid intima-media thickness in patients with non-alcoholic fatty liver disease: a population-based case-control study. Korean J Gastroenterol.

[28] Park HE (2016). Nonalcoholic Fatty Liver Disease Is Associated With Coronary Artery Calcification Development: A Longitudinal Study. J Clin Endocrinol Metab.

[29] Sung KC (2016). Fatty Liver, Insulin Resistance, and Obesity: Relationships With Increase in Coronary Artery Calcium Over Time. Clin Cardiol.

[30] Li Z (2014). Prevalence of nonalcoholic fatty liver disease in mainland of China: a meta-analysis of published studies. J Gastroenterol Hepatol.

[31] Clark JM (2006). The epidemiology of nonalcoholic fatty liver disease in adults. J Clin Gastroenterol.

[32] Lavoie JM, Pighon A (2012). NAFLD, Estrogens, and Physical Exercise: The Animal Model. J Nutr Metab.

[33] Gutierrez-Grobe Y (2010). Prevalence of non alcoholic fatty liver disease in premenopausal, posmenopausal and polycystic ovary syndrome women. The role of estrogens. Ann Hepatol.

[34] Kim MK (2015). Association between nonalcoholic fatty liver disease and coronary artery calcification in postmenopausal women. Menopause.

[35] Kim HJ (2016). Gender-based differences in the relationship between fatty liver disease and atherosclerosis. Cardiovasc J Afr.

[36] Liu H, Lu HY (2014). Nonalcoholic fatty liver disease and cardiovascular disease. World J Gastroenterol.

[37] Yang HR, Chang EJ (2016). Insulin resistance, body composition, and fat distribution in obese children with nonalcoholic fatty liver disease. Asia Pac J Clin Nutr.

[38] Erkan G (2014). The relationship between insulin resistance, metabolic syndrome and nonalcoholic fatty liver disease in non-obese non-diabetic Turkish individuals: A pilot study. Turk J Gastroenterol.

[39] Sung KC, Jeong WS, Wild SH, Byrne CD (2012). Combined influence of insulin resistance, overweight/obesity, and fatty liver as risk factors for type 2 diabetes. Diabetes Care.

[40] Korenblat KM, Fabbrini E, Mohammed BS, Klein S (2008). Liver, muscle, and adipose tissue insulin action is directly related to intrahepatic triglyceride content in obese subjects. Gastroenterology.

[41] Koroglu E (2016). Role of oxidative stress and insulin resistance in disease severity of non-alcoholic fatty liver disease. Turk J Gastroenterol.

[42] Blaha MJ (2011). The relationship between insulin resistance and incidence and progression of coronary artery calcification: the Multi-Ethnic Study of Atherosclerosis (MESA). Diabetes Care.

[43] Spahis, S., Delvin, E., Borys, J. M. & Levy, E. Oxidative Stress as a Critical Factor in Nonalcoholic Fatty Liver Disease Pathogenesis. *Antioxid Redox Signal* (2016). 10.1089/ars.2016.6776 27452109

[44] Bhatia LS, Curzen NP, Byrne CD (2012). Nonalcoholic fatty liver disease and vascular risk. Curr Opin Cardiol.

[45] Liu J (2012). Fatty liver, abdominal adipose tissue and atherosclerotic calcification in African Americans: the Jackson Heart Study. Atherosclerosis.

[46] Saarikoski, L. A. *et al*. Low serum adiponectin levels in childhood and adolescence predict increased intima-media thickness in adulthood. The Cardiovascular Risk in Young Finns Study. *Ann Med*, 1–26 (2016). 10.1080/07853890.2016.1226513 27534859

[47] Ntzouvani A (2016). Reduced circulating adiponectin levels are associated with the metabolic syndrome independently of obesity, lipid indices and serum insulin levels: a cross-sectional study. Lipids Health Dis.

[48] Hernaez R (2011). Diagnostic accuracy and reliability of ultrasonography for the detection of fatty liver: a meta-analysis. Hepatology.

[49] Kleiner DE (2005). Design and validation of a histological scoring system for nonalcoholic fatty liver disease. Hepatology.

[50] Hao Z (2016). The Association between Ideal Cardiovascular Health Metrics and Extracranial Carotid Artery Stenosis in a Northern Chinese Population: A Cross-Sectional Study. Sci Rep.

[51] Fan JG (2011). Guidelines for the diagnosis and management of nonalcoholic fatty liver disease: update 2010: (published in Chinese on Chinese Journal of Hepatology 2010; 18:163-166). J Dig Dis.

[52] Farrell GC (2007). Guidelines for the assessment and management of non-alcoholic fatty liver disease in the Asia-Pacific region: executive summary. J Gastroenterol Hepatol.

[53] Agatston AS (1990). Quantification of coronary artery calcium using ultrafast computed tomography. J Am Coll Cardiol.

[54] Luo TY (2016). Ideal Cardiovascular Health Metrics and Coronary Artery Calcification in Northern Chinese Population: A Cross-sectional Study. Biomed Environ Sci.

[55] Pletcher MJ, Tice JA, Pignone M, Browner WS (2004). Using the coronary artery calcium score to predict coronary heart disease events: a systematic review and meta-analysis. Arch Intern Med.

